# Current market rates for scholarly publishing services

**DOI:** 10.12688/f1000research.27468.1

**Published:** 2021-01-12

**Authors:** Alexander Grossmann, Björn Brembs

**Affiliations:** 1 Fakultät Informatik und Medien, HTWK Leipzig, Leipzig, Sachsen, 04277, Germany; 2 Institut für Zoologie - Neurogenetik, Universität Regensburg, Regensburg, Bavaria, 93053, Germany

**Keywords:** publishing, journals, costs, prices, scholarly publishing, scholarly communication, publisher

## Abstract

For decades, the supra-inflation increase of subscription prices for scholarly journals has concerned scholarly institutions. After years of fruitless efforts to solve this “serials crisis”, open access has been proposed as the latest potential solution. However, the prices for open access publishing are also high and are rising well beyond inflation. What has been missing from the public discussion so far is a quantitative approach to determine the actual *costs *of efficiently publishing a scholarly article using state-of-the-art technologies, such that informed decisions can be made as to appropriate *price *levels. Here we provide a granular, step-by-step calculation of the costs associated with publishing primary research articles, from submission, through peer-review, to publication, indexing and archiving. We find that these costs range from less than US$200 per article in modern, large-scale publishing platforms using post-publication peer-review, to about US$1,000 per article in prestigious journals with rejection rates exceeding 90%. The publication costs for a representative scholarly article today come to lie at around US$400. We discuss the additional non-publication items that make up the difference between publication costs and final price.

## Introduction

The affordability problem of scholarly publishing, i.e., the supra-inflationary price increases with stagnating library budgets, has been discussed for decades (see, e.g., 1– 7). In recent years, perhaps precipitated by some so-called ‘gold’ open access (OA) journals charging article-processing charges (APCs; fees usually charged to authors or their institutions upon acceptance for publishing an article and making it openly available), the *average cost of an article* has emerged as a useful measure with which to compare different business models (but see 8 for a critique). However, most authors refer to the *prices* charged by the publisher, not the actual *cost* to the publisher (e.g., 9– 12). One consequence of this mis-attribution is a potential overestimation of the actual costs of scholarly publishing due to the inclusion of the business models and pricing strategies of publishers into the calculation. To close this gap, here we provide a bottom-up calculation of the cost of efforts and services which are required to achieve a certain service level in order to publish an academic journal article. These calculations are analogous to what a new publisher would have to calculate before entering the publishing market. We compare our *cost* estimate with the current *pricing* schemes of publishers.

In this article, we assume the role of a newcomer to the academic publishing market and list the various steps and procedures for a representative publishing workflow according to current industry standards. Each step incurs a cost which can be determined by analyzing the market rates for each service or procedure. These costs comprise the direct costs. We also add several indirect (or fixed) cost items which do not accrue on a per article basis. The final per-article costs are then specified as a range depending on the number of articles published and the service level desired. These ranges denote current market rates at which customers can obtain publishing services.

## Methods

To arrive at a meaningful figure denoting how much the publication of an article *costs* on average, it is necessary to arrive at the exact cost for each step in the processing workflow of a manuscript being submitted for publication. These direct or variable costs then have to be combined with the indirect or fixed costs of running a publishing enterprise, such as staff costs, real estate, insurance and energy costs, etc. The former requires granular insight and expertise about the different service levels for the entire publishing workflow. The latter is commonly calculated as staff overhead. In this work, we have therefore calculated the cost for each step in the standard publication workflow under consideration of both fixed and variable costs. Both external and internal expenses have been taken into account as well as overhead costs to cover fixed non-direct company costs of the publishing venture.

### Direct or variable costs

Expenses and fees for each individual service have been arrived at from two main sources. Some standard services have been taken from openly available price lists ( Table 1).

**Table 1.** Publishing services and their fees.

Service Provider	Services	Permalink to fee page
CLOCKSS	Long-term preservation	https://perma.cc/2SQ2-VQUJ
CrossRef	DOI	https://perma.cc/N7BY-AJC3
Scholastica	Peer-review, publishing, typesetting	https://perma.cc/Z3DS-EZUW
Akron Aps	Peer-review management	https://perma.cc/U8J5-JS4E

Second, we requested quotes from vendors without publicly available fees, or turned to other sources ^13^. For services such as manuscript submission and peer review management systems we considered vendors such as Manuscript Central (Clarivate) and Editorial Manager (ARIES).

Other costs such as internal staff costs (including overhead, EU/US standard) were estimated taking into account not only current market costs we have requested ourselves, but also numbers from major publishing houses (MDPI, Wiley, Springer, DeGruyter, Frontiers, Ubiquity, SciELO, Open LIbrary of the Humanities). While some of these publishers have made their costs public ( Table 2), others have either provided their numbers under the condition of confidentiality or the numbers were gained from internal sources.

**Table 2.** Published itemized cost structures from publishers/service providers.

Publisher	Permalink to cost structure page
Frontiers	https://perma.cc/WKP4-R4D2
Open Library of the Humanities	https://perma.cc/9LEM-CDRL
Ubiquity	https://perma.cc/8U8K-AYZC
eLife	https://perma.cc/23GC-ARVB

For certain tasks, for example copyediting or typesetting, there are hundreds of individual companies worldwide providing those services on an industry-standard level. In our quote requests, we have considered only those with which we have collaborated in real business life so far or from which we know the performance and service level in detail from co-operations over two decades. Having compared the pricing of those service providers with others, we found only a very small variation of cost for such tasks, which justifies our practical approach. It was never our ambition to perform an exhaustive but always incomplete market study of service providers worldwide, but an attempt to provide an authoritative documentation of approximate current publishing costs as a valuable information tool for decision-makers and other stakeholders in policy drafting, contract negotiations or public discourse.

There are three main areas in which production steps have to be considered: content acquisition, content preparation (production) and content dissemination/archiving. Importantly, ‘content acquisition’ does not imply active acquisition of authors and/or manuscripts.

1. Content acquisition
  a. Searching and assigning reviewers
  b. Communication with reviewers
  c. Communication with authors
  d. Handling of re-submission process
  e. Plagiarism check
  f. Online submission system
  g. CrossRef Similarity Check
  h. CrossRef DOI for article
  i. CrossRef DOI for 2 or more reviews
  j. APC collection

2. Content preparation (production)
  a. Manuscript tracking system
  b. Production system check-in
  c. Technical checking of manuscript
  d. Copyediting
  e. Language editing
  f. Typesetting
  g. Formatting figures/graphs/tables
  h. Altmetric badge
  i. XML and metadata preparation
  j. Handling author corrections

3. Content dissemination/archiving
  a. Web OA platform and hosting
  b. CLOCKSS/Portico
  c. OAPEN
  d. Upload to Scopus, PMC, etc.

Pricing figures have been deducted by openly available price lists of vendors, as for example for Scholastica, Akron Aps, CrossRef, CLOCKSS (see Table 1, Table 2). In all other cases where pricing list or fees were not openly available on the web, prices were indicated after a direct request for proposal or communicated privately. For the latter we have checked with other partners to validate that information. Some service vendors have not split their services in a granular manner but offer a full service for more steps of the publishing workflow. In those cases, we have tried to split those costs or consider the full cost as part of one of the scenarios (see below) which cover the complete manuscript acquisition and article production process.

### Indirect or fixed costs

The calculation of per-article figures from costs that do not accrue on a per-article basis (e.g., salaries, annual fees, etc.) was based on the following assumptions: (i) The average STM article contains 12 printed pages ^12^. (ii) We estimated an average STM article to contain 10 non-text items such as figures or tables. (iii) We also assumed an average rejection rate of 50% after conventional (pre-publication) peer-review with at least two reports and ten contact requests to secure one reviewer. (iv) We assume a desk-rejection rate of 10% after editorial review. (v) We also base our staff costs on the granular workload per article and not on full-time equivalents (FTE). These assumptions entail that all editorial duties (on average 7.5 person-hours per submitted manuscript) are handled by in-house staff and none by academic editors, while peer-review is still performed by volunteer academics. In this way, staff costs, including overhead expenses, are calculated on a per-article basis (i.e., per published article, not per submitted manuscript). Salary costs are based on industry standards in more economically developed countries for the different editorial tasks. Overhead expenses can vary significantly depending on the profit and loss structure of the publisher and include rent, repairs, depreciation, interest, insurance, travel expenditures, labor burden, telephone bills, supplies, taxes, accounting fees, etc. We have estimated an average 33% overhead on top of salary costs. The following publication tasks are commonly covered by annual (membership) fees plus an initial, one-time set-up or installment fee: Web OA platform and hosting, CLOCKSS/Portico, OAPEN, Altmetric Badge and Crossref. Because these costs accrue regardless of how many articles are published (i.e., fixed costs), we have calculated per-article costs for journals with different numbers of articles published per year.

While some general fixed costs are covered by salary overheads (see above), we deliberately chose to not include certain fixed costs: Cost of sales have not been considered because for open access journals sales representatives are required no longer which have to negotiate renewals of subscriptions with libraries on an annual basis. We also excluded management costs as these are highly variable and in large publishers with many journals (and hence articles), per article costs of management are often negligible. We realize that this may be different for publishers which publish low-volume journals but with nevertheless highly paid executives (see *Discussion*). Because making an article public (i.e., ‘publishing’) is distinct from locking it behind a paywall, we have also not calculated the often very significant paywall costs. While innovation (or acquisition of innovative technologies) as well as branding and advertising/marketing are crucial for a company to succeed and thrive in a market in the long term, we have also not included these costs as they are not directly related to publishing scholarly articles. Such costs would include conference attendance, advertisement in print, online, social media and search platforms, as well as search engine optimization (SEO). Similarly, government relations (lobbying) may be considered a necessary expense for any business, but as it does not directly relate to the process of publishing academic papers, we did not include these costs in our calculations either. However, we do discuss the probable extent to which these non-publication costs may affect pricing.

### Scenarios

The motivation for the above assumptions was to combine a robust cost estimate (i.e., sourced from measurable time efforts and industry salaries) with an upper bound cost estimate which would come to lie above most academic-run journals. We also calculated a cost estimate for articles handled exclusively by volunteer academics. Prices for journals where volunteer and compensated editors cooperate, will hence fall between these two extremes.

With a modern, decentralized/federated platform providing publishing functionalities without journals, some of these steps become obsolete, while others remain relevant. Steps that may become obsolete include DOIs, long-term archiving such as CLOCKSS or Portico, indices such as Scopus. Relevant steps remaining are typesetting/copyediting, XML preparation, format conversion, plagiarism checks.

We have grouped the various combinations of tasks and publication options into six broad scenarios, for which we have calculated all associated publication costs ( Table 3). These scenarios correspond either to existing publishing options or to options that have been discussed in the literature. For each of the six scenarios, we have also calculated the same costs, but assuming a 90% rejection rate (see raw data file).

**Table 3.** Publishing scenarios for which detailed cost calculations have been performed.

**Scenario A**	Scholastica including ms submission, standard peer-review, tracking system, OA webpage, hosting
**Scenario A2**	Scenario A, but PPPR
**Scenario B**	Generic service providers, ms submission, standard peer-review tracking system; OA webpage, hosting
**Scenario B2**	Scenario B, but PPPR
**Scenario C**	Generic service providers for content preparation with online platform; without external submission, reviewing, and tracking system; with DOI; no external hosting/archiving; volunteer editors
**Scenario C2**	Scenario C, but Scholastica

Importantly, all costs are calculated per published article, i.e., a journal that publishes 1,000 articles per year has received 2,000 articles if their rejection rate is 50%. Our costs are calculated for the 1,000 published articles, not for the 2,000 submissions the journal has received.

All the data we have based our calculations on are available at Figshare (see *Underlying data*; DOI: 10.6084/m9.figshare.8118197) ^14^.

An earlier, non-peer-reviewed version of this article can be found on PeerJ (DOI: 10.7287/peerj.preprints.27809v1).

## Results

One of the first findings of our calculations is that in order to employ at least one 50% FTE of an in-house editor, a journal has to publish approx. 100 articles per year or more. Hence, in the following, we will base our estimates on journals publishing at least 100 articles per year (corresponding to 50% FTE) or 1,000 articles (corresponding to 5 FTEs), to show the spread of fixed and indirect costs over the number of articles published.

Our estimate of per-article publishing costs in a conventional pre-publication peer-review (50% rejection rate) scenario where all editorial duties are performed by in-house staff (Scenario B) ranges from US$643.61 for a journal that publishes 100 articles per year down to US$565.15 for such a journal that publishes 1,000 articles (or more, as the indirect costs become increasingly negligible around this value). These values consist of US$266.53 direct publishing costs (i.e., CrossRef Similarity Check, CrossRef DOI for an article, CrossRef DOI for two or more reviews, copyediting, typesetting, formatting figures/graphs/tables, altmetric badge, upload to Scopus and XML and metadata preparation), US$289.91 for editorial staff and US$8.72 to US$87.18 for 1,000 to 100 articles, respectively, in indirect costs (i.e., Web OA platform and hosting, CLOCKSS, OAPEN, Altmetric Badge and Crossref).

These numbers were calculated using generic, full-service providers (based in India), where applicable. There are open access service providers that provide packaged deals for the same services as these generic service providers. We have calculated the same steps using a well-known provider in this area, Scholastica (Scenario A). Interestingly, these figures are slightly higher: US$374.08 for direct publishing costs and US$5.92 to US$59.18 for 1,000 to 100 articles, respectively, for indirect costs (editorial staff costs remain the same).

While these costs have been calculated for a generic journal with 50% rejection rate, per-article costs will increase with increased rejection rates and decrease with less rejections as in, e.g., a post-publication peer-review (PPPR) model. In a journal that uses generic service providers and publishes all submitted manuscripts as PDF preprints with a DOI before performing otherwise identical peer-review as described above (i.e., PPPR with in-house editors and volunteer reviewers), per article editorial services drop from US$289.91 to US$140.69 (Scenario A2/B2), with all other costs remaining nearly identical. Conversely, prestigious journals with rejection rates of around 90% see their costs rise to US$1053.87 for 100 articles per year or US$770.53 for the larger journals with about 1,000 articles per year (generic service providers).

These numbers also show that for a conventional journal today, where academics perform their editorial duties on a volunteer basis (i.e., Scenario B, but no editorial costs as editor salaries are paid for by their academic institutions), direct publication costs come to lie at US$266.53 with generic service providers and total costs depend on the scale at which the journal operates. Small journals with 100 articles would face average per article total publication costs of US$353.71, while journals with 1,000 or more articles would only face costs of US$275.25 or less per published article. Even at the highest convenience for a small, volunteer-run journal, costs come to lie at US$454.63 where a full-service provider (Scholastica) handles all of the technical aspects of the work (Scenario C2).

The above calculations (summarized in Table 4) demonstrate economies of scale. The more articles are being published, the lower the costs for each article, approaching the fixed costs for each article.

**Table 4.** Different scenarios of journal organization, ordered by total per article costs (in US$). The scenarios are labeled with A, A2, B, B2, C, C2 (see Table 3).

scenario	total	direct	indirect	in-house staff
Conventional peer review, Scholastica, 100 articles (A)	**723.16**	374.08	59.18	289.91
Conventional peer review, Scholastica, 1,000 articles (A)	**669.90**	374.08	5.92	289.91
Conventional peer review, generic providers, 100 articles (B)	**643.61**	266.53	87.18	289.91
PPPR, Scholastica, 100 articles (A2)	**597.74**	369.88	87.18	140.69
Conventional peer review, generic providers, 1,000 articles (B)	**565.15**	266.53	8.72	289.91
PPPR, Scholastica, 1,000 articles (A2)	**519.28**	389.88	8.72	140.63
PPPR, generic providers, 100 articles (B2)	**469.32**	241.45	87.18	140.69
Volunteer editors, Scholastica, 100 articles (C2)	**454.63**	358.33	47.18	49.12
Volunteer editors, Scholastica, 1,000 articles (C2)	**412.16**	358.33	4.72	49.12
PPPR, generic providers, 1,000 articles (B2)	**390.86**	241.45	8.72	140.63
Volunteer editors, generic providers, 100 articles (C)	**237.35**	141.05	47.18	49.12
Volunteer editors, generic providers, 1,000 articles (C)	**194.89**	141.05	4.72	49.12

Because of the economies of scale and recent calls for the replacement of journals with a modern publishing platform ^15– 20^, we have also calculated the cost of publishing the annual output of the STM community, approx. 3 million articles, on such a platform that facilitates PPPR organized by academic editors on a single, decentralized, federated platform running modern software solutions. Such a platform would dispense with several production steps which are necessitated by the current balkanization of the literature in different journals published by different publishers, but keep others (see *Methods*). In this scenario, the indirect and fixed costs per article approach zero due to the high number of published articles (but see Discussion), such that the only remaining costs would be the direct publishing costs of US$190.17 per published article.

**Figure 1.**
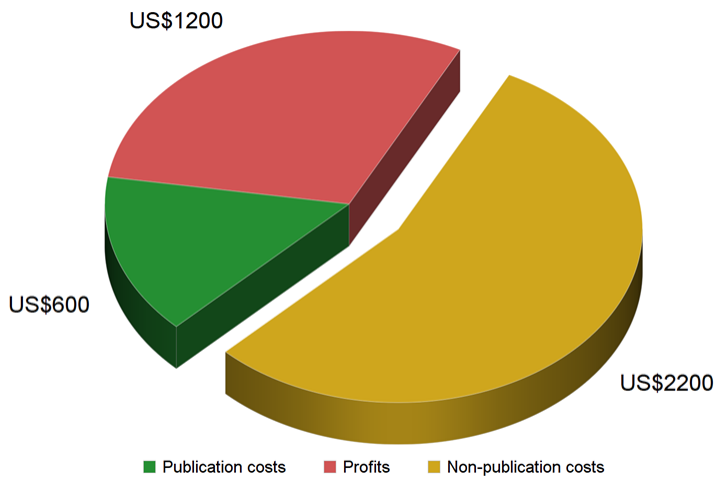
Subscription price and cost items. Assuming the commonly accepted US$4,000 price tag for a subscription article, published profit margins of 30% and our calculation of US$600 in publication costs for a full-service subscription article (scenario A/B, see Table 4), there remain US$2,200 in non-publication costs per article.

Finally, taking a ballpark cost figure of US$600 for a scholarly article with full editorial services (i.e., scenario A/B) and comparing it to the low end of the average price estimate for a subscription article of about US$4,000, it becomes clear that publication costs only cover 15% of the subscription price ( Figure 1). Assuming a conservative profit margin of 30% (i.e., US$1,200 per article) for one of the large publishers ^21– 24^, there remains a sizeable gap of about US$2,200 in non-publication costs, or 55% of the price of a scholarly subscription article ( Figure 1).

## Discussion

Since the 1990s, it has been recognized that the prices of scholarly journals were escalating at unsustainable rates ^3^. In the last 30 years, this “serials crisis” has never been coherently addressed, let alone solved. With this work, we aim to provide more financial evidence for future evidence-based policies addressing the affordability problem of scholarly communication ^1, 2^.

### Prices and costs

Not only current discussions are addressing the affordability problem in the unit of cost per article ^9– 12, 25– 28^ and we follow this precedent. Drawing from publicly available price lists and industry-standard service costs, we find that publishing costs per article vary from US$194.89 to US$723.16, depending on the level of service and publishing volume ( Table 4). It is important to note that these are conservative estimates, likely to constitute upper bounds, where innovation and changes in practice can be expected to decrease costs.

Perhaps not surprisingly, the convenience of outsourcing the main publishing services to a specialized full-service provider comes with a small increase in cost (scenario A vs. scenario B), when compared to an itemized sourcing of publishing services. In our cost estimate, we have not factored in the management cost of sourcing the itemized services, as we have not included company management in our calculations. Any decision between these two options will thus have to be made after factoring in such costs as well.

Even in the rare, most expensive case, these costs compare very favorably both to the current subscription pricing of around US$4,000–5,000 and current APCs (US$1,400–2,200). Our highest value encompasses conventional, journal-based pre-publication peer-review with a generic 50% rejection rate at a small journal (~100 articles per year) where all management of peer-review is performed by in-house editorial staff with no volunteer academic editors. Our data suggest that increasing only the rejection rate, for example from 50% to 90%, leads to an increase in publication costs of around 30–40% (e.g., in scenario B from US$565.15 to US$770.53 for 1,000 article journals or from US$643.61 to US$1,053.87 for 100 article journals). Apparently, this is a consequence of the respective increase of direct personnel expenses for managing the peer review process and communicating with both reviewers and authors for classical pre-publication peer review. As currently most highly selective journals publish on the order of 800–900 research articles per year about US$1,000 per article can be seen as an upper bound of total publication costs at such journals.

### Market rates for publishing services

The workflow we model consists of verifiable, modular components, available to any entity with the desire to enter the academic publishing world. Several publishers are already on the record to operate at similar costs to the ones we have calculated. These publishers include, but are not limited to SciELO, Pensoft/arpha, Open Library of the Humanities, Ubiquity, PeerJ or Scholastica. In fact, the 2018 STM report cites survey-based data that arrive at only slightly higher average costs than our calculation (US$420–650, excluding overhead, i.e., about US$560–870 with overhead) ^12^. Our calculations also fall in the same range as other methodologies ^29^.

Our calculations show that with publishing volumes exceeding 1,000 articles per year, fixed costs shrink below 1% of the direct article costs and become negligible. This was expected and already concluded in a previous analysis ^30^. These insights are important for designing a transition towards a scholarly publishing platform instead of journals.

Due to the limited possibility in dividing labor contracts into arbitrarily small portions, we find that journals with volumes below approx. 100 articles per year would be best served financially if they operated on the concept of volunteer academic editors handling the peer-review, instead of in-house staff.

In conclusion, given the congruence of the available data and the publicly available prices for the services required, the market rate ranges for publication services we arrive at here do not appear controversial. Perhaps more controversial is the number and amount of non-publication costs a scholarly article, funded by the taxpayer, ought to contain.

### Non-publication costs

If the lowest publication costs for journals with volunteer editors constituted merely 5–10% of current subscription prices and publicly reported publisher profits only amount to an additional 30–40%, which non-publication costs are publishers currently facing and taxpayers paying for? While these costs are opaque and variable between publishers and, indeed, between journals, some estimates can be made from publicly available data. If one assumes revenue of about US$4,000 per subscription article (i.e., on the low end of the converging estimates), a conservative 30% profit margin (i.e., US$1,200 per article) for one of the large publishers ^21– 24^ and generous publication costs of US$600 per article (scenario A/B; Table 4), then there remains a sizeable gap of about US$2,200 in non-publication costs per article - more than the sum of publication costs and profits combined, or 55% of the subscription cost of a scholarly article ( Figure 1). While some of these costs may be considered necessary for any business, none of them are associated with publishing primary research articles (see *Methods*).

***Running a business: Management*.** While our cost calculations include generic running costs such as rent, repairs, depreciation, interest, insurance, travel expenditures, labor burden, telephone bills, supplies, taxes, accounting fees, etc., we have explicitly omitted some indirect costs such as management cost and paywalls. For instance, according to their 2016 tax statement, the New England Journal of Medicine spends 4% of its publication revenue on their top ten management staff alone (which would translate to about US$160 per article if applied to our example above; Figure 1).

***Preventing access: Paywalls*.** Subscription journals also face costs associated with paywalls. It’s difficult to estimate the cost of such technology for publishers, but the cost of a new paywall for the New York Times was reported to lie between US$25–50 million ^31, 32^. Alternatively, as the functional distinction between subscription articles and OA articles is precisely the missing paywall in OA articles, one could also assume that publishers arrive at their current APC pricing of around US$2,000 by subtracting paywall costs from their subscription price. This assumption would entail paywall costs of approx. US$2,000 per article (i.e., the difference between APC and subscription pricing).

On top of the technical costs of a paywall, one may also consider the legal fees for defending paywalls for this cost item. Publishers have a track record of litigation with regard to articles outside of their paywalls and regularly seek damages in court for actual or perceived threats to their subscription business model ^33– 39^. These costs accrue by seeking to enclose the scholarly literature within the paywalls of publisher via alternative routes in addition to the digital paywalls.

***News, advertising, sales, marketing, public relations: branding*.** Another cost item is publishing non-research content. For instance, for 2017, PubMed lists a total of 1,595 articles published by the Lancet, while Clarivate Analytics only counts 302 articles for their Impact Factor. Assuming that only the latter articles amount to primary research publications, this journal’s revenue also pays for 1,293 non-research articles. Similar numbers also hold for other prestigious journals (e.g.: Nature: 837/2469, Science: 769/2629, New England Journal of Medicine: 327/1449; research/total), often with their own journalist and editorial staff commissioning articles and/or reporting themselves on research and policy news. However, the number of journals where this can constitute a significant fraction of their total costs is presumably small, likely restricted to the most prestigious journals.

Prestigious journals also often practice active author or materials acquisition by traveling to conferences and laboratories, building networks in a strategy to entice the next exciting research finding to be published in their journals. Active author acquisition accrues costs both in terms of travel and time spent networking and communicating with authors that is not covered in our cost estimates (see *Methods*).

Sometimes, new journals also need to engage in such author acquisition practices, which, perhaps, can be best subsumed under general marketing or public relations costs required for building and maintaining a brand. These marketing costs also include, e.g., advertising in various venues targeting both authors and subscribers. For many publishers it is also common to promote their brand at conferences and institutions with, e.g., hosted speakers, travel grants or sponsored awards.

Because of the complex, time-consuming negotiations with libraries on ever tighter budgets due to the supra-inflationary subscription price increases, publishers also need to employ expert sales teams. The task of these sales teams is not only to find the most irresistible way to package and bundle subscription journals and/or databases, but also to device the most inexorable psychological strategy for their negotiations with librarians. These sales teams need to operate in close connections with the various advertising, marketing and public relations teams of the publisher to accomplish a coherent brand image. One may argue that in times of OA, these sales costs are not necessary expenses any more and more associated with paywall costs than with publication costs. On the other hand, in an OA world, one may argue that branding was never more important for author acquisition.

***New technologies: innovation and acquisitions*.** Publishers also need to invest in innovation in order to stay current with their technologies and functionalities. While scholarly publishers have been quick to transition from print to web-based technologies in the past, the digital functionalities of most of the scholarly literature today lag at least a decade behind current functionalities of other digital objects outside of the scholarly literature. The level of investment in innovation thus remains unclear and its effects questionable. Instead of investments into their own technological innovation, publishers today appear to acquire companies that have invented desired functionalities around the scholarly workflow, with the goal to provide services beyond publications ^40– 43^.

***Government relations: Lobbying*.** Most international publishers, as any other corporation, also spend significant amounts of money on government relations (i.e., lobbying). Some of these corporations employ staff at the vice president level not only in the most important research nations, but also at the level of supra-national bodies such as the European Commission ^44^. These staff, in turn, employ assistants and other members of their teams. Obviously, the task of these employees is to protect current revenue streams, e.g., subscription or APC income. For instance, one publisher, Elsevier, spends more than 400,000€ per year on lobbying at the level of the European Commission alone ^45^. The consequences of such efforts have been observable, e.g., in the so-called “Finch Report” in the UK ^46^, which surprised many commentators with its publisher-friendly recommendations ( 44, see, e.g., 47).

***Which non-publication costs should remain bundled up with publishing?*** Regardless of all of these estimates necessarily remaining vague and imprecise, the fact remains that the scholarly community must eventually make a number of decisions if it is to tackle the affordability problem. Which of the above non-publication costs should remain bundled up with the process of publishing scholarly research articles? Which of these costs are avoidable, which necessary and which even desirable? Are profit margins of 30–40% on taxpayer funds tolerable?

In fact, one may even ask whether many of the services we list as part of the scholarly publishing standard are actually necessary for scholarly publishing. After all, journals such as the Journal of Machine Learning Research, Discrete Analysis or the Journal of Open Source Software publish their articles with internal costs below US$10 ^48, 49^. Likewise, the preprint archive arXiv publishes their articles at similar costs ^50^.

## Data availability

### Underlying data

Figshare: Journal_Production_Cost_010519.xlsx. https://doi.org/10.6084/m9.figshare.8118197.v1 ^14^.

This project contains the data used to calculate production costs for articles.

Data are available under the terms of the Creative Commons Attribution 4.0 International license (CC-BY 4.0).
