# Supplementary material for: Current market rates for scholarly publishing services
Source: F1000Res. 2021 Jul 1;10:20. Originally published 2021 Jan 12. [Version 2] doi: 10.12688/f1000research.27468.2 (PMC8276192; doi:10.12688/f1000research.27468.2)
Supplement: Peer review history

# Peer review: Reviewer response for version 2

**DOI:** 10.5256/f1000research.57214.r88867

**Published:** 2021-07-12

**Authors:** Pandelis Perakakis

**Affiliations:** 1 Open Scholar CIC, Birmingham, UK; 2 Complutense University of Madrid, Madrid, Spain

All initial concerns and recommendations were addressed in the revised version of the manuscript.

Is the work clearly and accurately presented and does it cite the current literature?

Partly

If applicable, is the statistical analysis and its interpretation appropriate?

Not applicable

Are all the source data underlying the results available to ensure full reproducibility?

Yes

Is the study design appropriate and is the work technically sound?

Yes

Are the conclusions drawn adequately supported by the results?

Yes

Are sufficient details of methods and analysis provided to allow replication by others?

Yes

Reviewer Expertise:

Scholarly communication, Open peer review

I confirm that I have read this submission and believe that I have an appropriate level of expertise to confirm that it is of an acceptable scientific standard.

# Peer review: Reviewer response for version 2

**DOI:** 10.5256/f1000research.57214.r88866

**Published:** 2021-07-08

**Authors:** Lisa Rose-Wiles

**Affiliations:** 1 Seton Hall University Libraries, Seton Hall University, South Orange, USA

Is the work clearly and accurately presented and does it cite the current literature?

Partly

If applicable, is the statistical analysis and its interpretation appropriate?

Not applicable

Are all the source data underlying the results available to ensure full reproducibility?

Partly

Is the study design appropriate and is the work technically sound?

Yes

Are the conclusions drawn adequately supported by the results?

Yes

Are sufficient details of methods and analysis provided to allow replication by others?

Yes

Reviewer Expertise:

Library science, information literacy, citation analyses, circulation and journal/book pricing analyses.

I confirm that I have read this submission and believe that I have an appropriate level of expertise to confirm that it is of an acceptable scientific standard.

# Peer review: Reviewer response for version 1

**DOI:** 10.5256/f1000research.30360.r77201

**Published:** 2021-02-16

**Authors:** Lisa Rose-Wiles

**Affiliations:** 1 Seton Hall University Libraries, Seton Hall University, South Orange, USA

I concur that the “supra-inflation increase of subscription prices for scholarly journals” is a critical issue, especially for academic libraries, and that this is a valuable contribution to the literature.

 I also concur with the previous reviewer that the “scenarios” and tables could be better explained and organized for greater clarity. Also further indicators of variance would be helpful - for example, there are surely significant differences (especially in “fixed or indirect costs”) based on geographic location. I also find myself wondering which (if any) of the costs to publishers are tax deductible, which would effectively increase the profit margin.

 While the main focus of the manuscript is the cost of producing articles, to place this in broader perspective It would be helpful to clarify more explicitly that there are two major price components to journal articles: (1) Journal subscription costs, which are typically borne by libraries (although no doubt some researchers still subscribe to their favorite journals themselves), and (2) article-processing charges (APC’s) which are in principle an open-access alternative to subscriptions, but in practice often simply shift the cost from an institution’s library to its faculty, research office and/or some other institutionally-funded entity. Both contribute directly or indirectly to the high price of student tuition and the chronic under-funding of many academic libraries, but subscription prices seem to be the greater issue since the average “per article price” (see below) is considerably higher. 

 For readers not necessarily familiar with the various journal pricing models, the authors might note that both journal prices and APC’s vary enormously by discipline, especially STEM vs. humanities, and that the practice of “Big Deal” bundling (especially by large commercial publishers) can make it very difficult for librarians to disentangle the actual subscription cost per journal. It would be helpful to include some discussion and more explicit data on the widely varying range of APC’s, which, in my experience, seem independent of actual subscription costs. I am more familiar with the pricing models for STEM journals (typically > US $2,500 APC regardless of journal subscription price), but it is my impression that when humanities journals do charge authors, the fee is much lower. This would be an interesting topic to explore in a follow-up article. Granted that STEM articles typically include multiple tables and figures that are costly to produce, but is the price differential really as substantial as the differences in subscription costs or APC’s?

 In the final paragraph of the results (p.6) and in Figure 1, the authors compare the article cost with “the average price estimate for a subscription article of about US$4,000”. Where did this figure come from and what is it based on? Is this an across the board “per library” average, in which case the wide variation in prices paid by different libraries (at least in the US) is surely another confounding factor. Again, there should be some discussion of the vast difference in pricing among disciplines, or if this is based on STEM journals as I suspect, that should be clarified. Also the average APC price should be included in the comparison; it is interesting that it appears to be considerably less than the quoted US$4,000 subscription “per article” cost. This brings up a further question for the future: in hybrid journals, does charging APC’s result in lower subscription prices, or are APC’s effectively layered on top of subscription prices?

 Another point that is only tangentially referenced (e.g. “volunteer academic editors”) is that many of the costs associated with publishing are actually borne by the institution and its academic faculty, who not only produce the scholarship but also review articles and often act as editors as well. This labor is typically unpaid. “Volunteer academic editors” in well-funded institutions may receive some form of course release, additional office space and/or clerical support, but again (see first paragraph) these costs are borne by the institution and to some degree passed on to its students; they are not borne by the publishers. Of course this point has been made many times before, but since the authors are discussing various costs involved in publication it is worth noting again.

 As a final comment, it would be informative to have responses from publishers, especially small “non-profit” or “not-for-profit” publishers on this publication.

 Very thought provoking!

Is the work clearly and accurately presented and does it cite the current literature?

Partly

If applicable, is the statistical analysis and its interpretation appropriate?

Not applicable

Are all the source data underlying the results available to ensure full reproducibility?

Partly

Is the study design appropriate and is the work technically sound?

Yes

Are the conclusions drawn adequately supported by the results?

Yes

Are sufficient details of methods and analysis provided to allow replication by others?

Yes

Reviewer Expertise:

Library science, information literacy, citation analyses, circulation and journal/book pricing analyses.

I confirm that I have read this submission and believe that I have an appropriate level of expertise to confirm that it is of an acceptable scientific standard, however I have significant reservations, as outlined above.

# Response

**Published:** 2021-05-25

**Authors:** Björn Brembs

**Affiliations:** Institute of Zoology - Neurogenetics, Universität Regensburg, Germany

**Response to Reviewer #2: Lisa Rose-Wiles:**

*I also concur with the previous reviewer that the “scenarios” and tables could be better explained and organized for greater clarity.*

**We thank both reviewers for their suggestions and are confident to have now adequately addressed this very valid concern in the new, revised version of the manuscript.**

*Also further indicators of variance would be helpful - for example, there are surely significant differences (especially in “fixed or indirect costs”) based on geographic location. I also find myself wondering which (if any) of the costs to publishers are tax deductible, which would effectively increase the profit margin.*

**We have now further emphasized that our salary calculations are based on western (i.e., US/EU) industry standards and that countries with lower salary levels would hence face lower costs. This is to arrive at upper bound figures, describing the costliest way of publishing, from which one can always find ways to reduce costs. We have further emphasized this approach also at the very beginning of the Discussion section now.**

*While the main focus of the manuscript is the cost of producing articles, to place this in broader perspective It would be helpful to clarify more explicitly that there are two major price components to journal articles: (1) Journal subscription costs, which are typically borne by libraries (although no doubt some researchers still subscribe to their favorite journals themselves), and (2) article-processing charges (APC's) which are in principle an open-access alternative to subscriptions, but in practice often simply shift the cost from an institution's library to its faculty, research office and/or some other institutionally-funded entity. Both contribute directly or indirectly to the high price of student tuition and the chronic under-funding of many academic libraries, but subscription prices seem to be the greater issue since the average “per article price” (see below) is considerably higher.*

*For readers not necessarily familiar with the various journal pricing models, the authors might note that both journal prices and APC's vary enormously by discipline, especially STEM vs. humanities, and that the practice of “Big Deal” bundling (especially by large commercial publishers) can make it very difficult for librarians to disentangle the actual subscription cost per journal. It would be helpful to include some discussion and more explicit data on the widely varying range of APC's, which, in my experience, seem independent of actual subscription costs. I am more familiar with the pricing models for STEM journals (typically > US $2,500 APC regardless of journal subscription price), but it is my impression that when humanities journals do charge authors, the fee is much lower. This would be an interesting topic to explore in a follow-up article. Granted that STEM articles typically include multiple tables and figures that are costly to produce, but is the price differential really as substantial as the differences in subscription costs or APC's?*

**We agree with all the arguments here. Therefore, we now more explicitly reference the longer, preprint version of our manuscript, where we not only discuss pricing strategies and business models, as suggested, but also policy options. However, in this more condensed version, we made a conscious effort to focus exclusively on the cost aspect, as we felt the original version was too long, unwieldy and difficult to read as it was covering too many topics at the same time.**

*In the final paragraph of the results (p.6) and in Figure 1, the authors compare the article cost with “the average price estimate for a subscription article of about US$4,000”. Where did this figure come from and what is it based on? Is this an across the board “per library” average, in which case the wide variation in prices paid by different libraries (at least in the US) is surely another confounding factor.*

**This explanation was indeed lost as we transitioned from our longer preprint version to this shorter one. We are very grateful for having this pointed out. We now reference the sources of these calculations. The most recent one is very simple: dividing the estimated US$10b in journal revenue by the number of published articles (2m): ~5k. As some other sources mention 4k, we went with the lower bound of these estimates in the literature.**

*Again, there should be some discussion of the vast difference in pricing among disciplines, or if this is based on STEM journals as I suspect, that should be clarified. Also the average APC price should be included in the comparison; it is interesting that it appears to be considerably less than the quoted US$4,000 subscription “per article” cost. This brings up a further question for the future: in hybrid journals, does charging APC's result in lower subscription prices, or are APC's effectively layered on top of subscription prices?*

**We now mention the APC price range of US$1,400-2,200 and the 8 references upon which we base this range.**

*Another point that is only tangentially referenced (e.g. “volunteer academic editors”) is that many of the costs associated with publishing are actually borne by the institution and its academic faculty, who not only produce the scholarship but also review articles and often act as editors as well. This labor is typically unpaid. “Volunteer academic editors” in well-funded institutions may receive some form of course release, additional office space and/or clerical support, but again (see first paragraph) these costs are borne by the institution and to some degree passed on to its students; they are not borne by the publishers. Of course this point has been made many times before, but since the authors are discussing various costs involved in publication it is worth noting again.*

**We now make it more explicit what is being paid in terms of salary and which work is being carried out by volunteers. We also explicitly mention the costs covered by institutions (e.g., salaries, servers) and relate them to per-article cost.**

*As a final comment, it would be informative to have responses from publishers, especially small “non-profit” or “not-for-profit” publishers on this publication.*

**We list numerous publishers which are already on the record for publishing in this cost range and cite journals that have much lower costs than our figures. In the acknowledgements, we mention that we have been made privy of internal cost structures to validate and test our figures. As we mention in the article, our numbers match those from other methodologies (e.g. surveys). The preprint version has been available since 2019 and has even received attention in trade organs such as the “Scholarly Kitchen”. In other words, publishers have already vetted our numbers and have had ample opportunity over a number of years to comment on our work and improve it by criticism. Given that our numbers match so well with the available evidence and that our article has received considerable attention from the industry since 2019, perhaps one may interpret the fact that the expensive legacy publishers so far have not publicly commented on these numbers, as evidence that there may not be much left to criticize? Of course, another reason may be that the more expensive publishers may hesitate to draw more attention to the fact that they are so much more expensive. In this case, we would appreciate any suggestions in how we may be able to force these publishers to acknowledge and comment on our calculations. F1000Research allows for comments on the manuscript and perhaps a drive to invite publishers of all ranges to contribute so such a comment may be instructive?**

**Society publishers, so far, have refrained from public comments other than generally stating that they use publication income to finance society services. We allude to many such and related costs in the “non-publication costs” section of the manuscript.**

# Peer review: Reviewer response for version 1

**DOI:** 10.5256/f1000research.30360.r77204

**Published:** 2021-02-01

**Authors:** Pandelis Perakakis

**Affiliations:** 1 Open Scholar CIC, Birmingham, UK; 2 Complutense University of Madrid, Madrid, Spain

This article reports a breakdown of article publication costs —from manuscript submission to indexing— for different publication scenarios. There are at least two fundamental reasons why analysing and reporting the realistic cost of publishing a research article is of paramount importance. First, given the exorbitant sums of public money spent to publish the world’s scholarly output it is imperative that we know where exactly this money goes. This information can perhaps mobilise governments, funders, individual scholars, and the general public to demand more sustainable publication models that provide only those essential services that truly add value to scholarly works. Second, a granular account of publishing costs can incentivise more scientific societies and academic groups to abandon their commercial publishers and establish alternative models that better serve the needs of scholarly communication. There is no doubt therefore that this is an extremely valuable report that should be widely disseminated. My review will mainly focus on some recommendations that in my opinion would improve the presentation and utility of the results.

 The cost analysis provided in this report is based on a list of 24 services grouped in three distinct categories: content acquisition, preparation, and dissemination. A first minor observation is that in the category of content acquisition, it would probably be more intuitive to list “online submission system” as the first item (before “searching and assigning reviewers”) to match the publication workflow. A second and more important observation is that in this list, and throughout the manuscript, services are sometimes confounded with service providers. Since there are more than one providers for each of the services, I strongly recommend that the list only includes services, whereas in the manuscript different options for each service can be discussed. For example Crossref is not the only option for “DOI registration”, which I believe should be the title of this particular service. Similarly, the service provided by CLOCKSS/LOCKSS/Portico, could be called “long-term digital preservation”. Also, it is not clear what the service provided by OAPEN is, while “upload to Scopus…”, could be called “distribution to indexing services”. Should the authors decide to follow this recommendation I would advise to also modify the accompanying excel file accordingly.

 The “Direct or variable costs” subsection in the methods section would be easier to follow if it was structured differently, starting with the list of services and then explaining how pricing information was obtained.

 Following the logic that the list of services is the nucleus of the cost analysis, and the report itself, I suggest that table 1 is modified so that the first column displays the services and the second column the providers. Also, it is important that the terms in the services list are used consistently throughout the manuscript. For example, in table 1 we find a service termed “peer review” and another called “peer review management”. It is not clear how these services correspond to the original list. When a service provider offers more than one service in the list, I would recommend to include all of these services in the table. If it is considered convenient to create a new grouping (e.g., peer review management), it should be clear —in the table and in the manuscript— which services from the original list are included in this group (e.g., 1a, 1b, 1c, etc.).

 The “scenarios” section should include a more thorough description of the different scenarios, as well as the motivation for selecting each of these scenarios as a separate use case. This description of what each scenario refers to is attempted in the “results” section but in a rather anarchic and hard to follow manner. In the “scenarios” section the authors vaguely mention that these scenarios “...correspond either to existing publishing options or to options that have been discussed in the literature”, but neither are these options clearly delimited nor specific references are provided. I therefore found it very hard by reading the manuscript to understand exactly which publication model, combination of services, and choice of providers each scenario refers to. This was made possible only by carefully analysing the formulas for the different calculations in the excel file. However, even after this more careful analysis, the motivation behind each scenario remained unclear. For example, it seems that the only difference between scenarios A and B in terms of content acquisition is using one service provider instead of another. In scenario A scholastica is used for online submission management (cell L18), while Akron is used in scenario B (cell J19).

 I recommend that the different scenarios are not defined based on the choice of specific providers but according to different, clearly explained publication models, characterised, for example, by use of existing publishing infrastructure (e.g., institutional or disciplinary repositories as in the case of existing overlay journals), variable rejection rates, number of published articles, review policies, voluntary or hired editorial work, etc. The available choice of providers for specific services (or groups of services) and their impact on the costs can be discussed in the manuscript and even presented as a separate table. However, I consider it important that the initial scenarios are reported without any reference to specific providers but rather using average (or low/high) estimates. Otherwise, in their present form, tables 3 and 4 are very hard to follow. In both tables publication choices (number of articles, review models, voluntary editors) are mixed with providers (scholastica, generic providers, etc.) without a clear description (in the table or the manuscript) of the different publication models, or the services offered by each provider. For example, from the excel file I deduced that scenarios A2, B2 and C do not include costs for online submission, but it is not clear from the discussion in the manuscript or from the brief labels in table 3, which exact models allow the omission of these costs and how.

 Insisting on the necessity to adequately describe the different publication scenarios, in the “scenarios” section the authors refer to a “decentralized/federated platform providing publishing functionalities”. However, again it is not clear what are the foundations of this model (e.g., a reference could be provided to the next generation repositories initiative promoted by COAR [1], or other similar proposals in the literature, such as in reference “20” in the manuscript), and whether this model is represented in one of the scenarios. Similarly, the authors briefly mention a PPPR model in the “results” section but there is no clear description of what exactly this model entails and which services from the original list allows to omit or circumvent.

 To summarise, I strongly recommend that the different publication scenarios refer to publication options (not choice of providers) and that they are concisely described in the corresponding section with references to the literature or to existing examples when possible. It should be clear which services from the list correspond to each scenario and how different scenarios allow the omission of certain services. The different options for service providers should be discussed separately. For example, it would be useful to report the impact on the publication costs of choosing scholastica or Akron as a provider for a specific list of services (drawn from the initial services list) in a given publication scenario.

 As a minor comment, I suspect that J21 is missing from the formula used to calculate cell J26. I would recommend that the authors had another careful look at their formulas to avoid similar omissions or errors.

 Overall, I commend the authors for their work and invite them to consider my recommendations that I believe will significantly improve the uptake of this extremely valuable information.

 References:

 [1] https://www.coar-repositories.org/news-updates/what-we-do/next-generation-repositories/

Is the work clearly and accurately presented and does it cite the current literature?

Partly

If applicable, is the statistical analysis and its interpretation appropriate?

Not applicable

Are all the source data underlying the results available to ensure full reproducibility?

Yes

Is the study design appropriate and is the work technically sound?

Yes

Are the conclusions drawn adequately supported by the results?

Yes

Are sufficient details of methods and analysis provided to allow replication by others?

Yes

Reviewer Expertise:

Scholarly communication, Open peer review

I confirm that I have read this submission and believe that I have an appropriate level of expertise to confirm that it is of an acceptable scientific standard, however I have significant reservations, as outlined above.

# Response

**Published:** 2021-05-25

**Authors:** Björn Brembs

**Affiliations:** Institute of Zoology - Neurogenetics, Universität Regensburg, Germany

**Response to Reviewer #1: Pandelis Perakakis:**

*The cost analysis provided in this report is based on a list of 24 services grouped in three distinct categories: content acquisition, preparation, and dissemination. A first minor observation is that in the category of content acquisition, it would probably be more intuitive to list “online submission system” as the first item (before “searching and assigning reviewers”) to match the publication workflow.*

**Thank you, corrected.**

*A second and more important observation is that in this list, and throughout the manuscript, services are sometimes confounded with service providers. Since there are more than one providers for each of the services, I strongly recommend that the list only includes services, whereas in the manuscript different options for each service can be discussed. For example Crossref is not the only option for “DOI registration”, which I believe should be the title of this particular service. Similarly, the service provided by CLOCKSS/LOCKSS/Portico, could be called “long-term digital preservation”. Also, it is not clear what the service provided by OAPEN is, while “upload to Scopus…”, could be called “distribution to indexing services”. Should the authors decide to follow this recommendation I would advise to also modify the accompanying excel file accordingly.*

**This was our oversight and it is an excellent suggestion. We are now following it 100%, also in the Excel spreadsheet.**

*The “Direct or variable costs” subsection in the methods section would be easier to follow if it was structured differently, starting with the list of services and then explaining how pricing information was obtained.*

**This is how it should be and we have no good explanation for why we did not write it that way. We have now re-ordered all components as suggested.**

*Following the logic that the list of services is the nucleus of the cost analysis, and the report itself, I suggest that table 1 is modified so that the first column displays the services and the second column the providers.*

**Corrected.**

*Also, it is important that the terms in the services list are used consistently throughout the manuscript. For example, in table 1 we find a service termed “peer review” and another called “peer review management”. It is not clear how these services correspond to the original list. When a service provider offers more than one service in the list, I would recommend to include all of these services in the table. If it is considered convenient to create a new grouping (e.g., peer review management), it should be clear -in the table and in the manuscript- which services from the original list are included in this group (e.g., 1a, 1b, 1c, etc.).*

**I see the point here, of course. We have been able to quickly fix the instances where it was just a matter of replacing a few words. However, it was not easy to address this very relevant point everywhere, as it is many times easier to refer to a series of steps with a shorthand rather than with a long list. Sometimes that shorthand can be a provider (e.g., Scholastica), sometimes it is a functionality such as, e.g., “OJS” for a system that manages submission, manuscript tracking and review management all at the same time. We have tried to rephrase as many of such instances as possible, but we are not sure if we were able to address this point in every single instance.**

*The “scenarios” section should include a more thorough description of the different scenarios, as well as the motivation for selecting each of these scenarios as a separate use case. This description of what each scenario refers to is attempted in the “results” section but in a rather anarchic and hard to follow manner. In the “scenarios” section the authors vaguely mention that these scenarios “...correspond either to existing publishing options or to options that have been discussed in the literature”, but neither are these options clearly delimited nor specific references are provided. I therefore found it very hard by reading the manuscript to understand exactly which publication model, combination of services, and choice of providers each scenario refers to. This was made possible only by carefully analysing the formulas for the different calculations in the excel file. However, even after this more careful analysis, the motivation behind each scenario remained unclear. For example, it seems that the only difference between scenarios A and B in terms of content acquisition is using one service provider instead of another. In scenario A scholastica is used for online submission management (cell L18), while Akron is used in scenario B (cell J19).*

*I recommend that the different scenarios are not defined based on the choice of specific providers but according to different, clearly explained publication models, characterised, for example, by use of existing publishing infrastructure (e.g., institutional or disciplinary repositories as in the case of existing overlay journals), variable rejection rates, number of published articles, review policies, voluntary or hired editorial work, etc. The available choice of providers for specific services (or groups of services) and their impact on the costs can be discussed in the manuscript and even presented as a separate table. However, I consider it important that the initial scenarios are reported without any reference to specific providers but rather using average (or low/high) estimates. Otherwise, in their present form, tables 3 and 4 are very hard to follow. In both tables publication choices (number of articles, review models, voluntary editors) are mixed with providers (scholastica, generic providers, etc.) without a clear description (in the table or the manuscript) of the different publication models, or the services offered by each provider. For example, from the excel file I deduced that scenarios A2, B2 and C do not include costs for online submission, but it is not clear from the discussion in the manuscript or from the brief labels in table 3, which exact models allow the omission of these costs and how.*

**It is completely obvious how this section must be confusing for readers and, again, we do not have a good explanation for why we did not provide sufficient detail. Our lack of explanation could also be seen in some of Lisa Rose-Wiles’ comments. In attempting to follow these suggestions, we now have provided not only detailed explanations for each scenario in the text, but have also updated Table 3. In brief, scenario A and B differ in that B cases source multiple, generic publishing providers for the different steps (lower cost, more contracts, requires expertise), while A sources many steps from a single provider, specialized in scholarly publishing (more convenient, less expertise required, higher cost). Scenario C also covers all steps in principle, but does not count some costs such as editors (volunteers) or servers (institutional server) or a submission/tracking system (e.g., OJS). Post-publication peer-review decreases rejections and hence price (A2/B2). Scenario C2 replaces, e.g., servers and OJS with Scholastica.**

*Insisting on the necessity to adequately describe the different publication scenarios, in the “scenarios” section the authors refer to a “decentralized/federated platform providing publishing functionalities”. However, again it is not clear what are the foundations of this model (e.g., a reference could be provided to the next generation repositories initiative promoted by COAR [1], or other similar proposals in the literature, such as in reference “20” in the manuscript), and whether this model is represented in one of the scenarios. Similarly, the authors briefly mention a PPPR model in the “results” section but there is no clear description of what exactly this model entails and which services from the original list allows to omit or circumvent.*

**We now cite Perakakis et al. 2010 very prominently, as well as the COAR report, together with a brief explanation of the concept. Both are referenced with the name they gave these solutions, i.e., “global open archive” or ”next generation repository”, respectively.**

*To summarise, I strongly recommend that the different publication scenarios refer to publication options (not choice of providers) and that they are concisely described in the corresponding section with references to the literature or to existing examples when possible. It should be clear which services from the list correspond to each scenario and how different scenarios allow the omission of certain services. The different options for service providers should be discussed separately. For example, it would be useful to report the impact on the publication costs of choosing scholastica or Akron as a provider for a specific list of services (drawn from the initial services list) in a given publication scenario.*

**We are confident that the current version now addresses all the raised issues and that our choice of scenarios becomes clearer in the way we explain them now.**

*As a minor comment, I suspect that J21 is missing from the formula used to calculate cell J26. I would recommend that the authors had another careful look at their formulas to avoid similar omissions or errors.*

**We have triple-checked these cells in the spreadsheet and could not find any missing values. We have also checked all adjacent cells for possible errors and were not able to find any. We have gone over the entire spreadsheet both for the consistency of the calculations, the accuracy of the text descriptions and whether any of the costs needed to be updated due to change market rates. We have not been able to locate any problems. We suggest arranging an online call with video and screen sharing to identify and remove potential errors we may have missed or are unable to identify.**
